# Satellite Positioning Accuracy Improvement in Urban Canyons Through a New Weight Model Utilizing GPS Signal Strength Variability

**DOI:** 10.3390/s25154678

**Published:** 2025-07-29

**Authors:** Hye-In Kim, Kwan-Dong Park

**Affiliations:** 1 PP-Solution Inc., 606 Seobusaet-gil #B-2311, Seoul 08504, Republic of Korea; hikim@ppsol.com; 2 Department of Geoinformatic Engineering, Inha University, 100 Inha-ro, Incheon 22212, Republic of Korea

**Keywords:** GPS, SNR, multipath, weight model, urban canyons

## Abstract

Urban environments present substantial obstacles to GPS positioning accuracy, primarily due to multipath interference and limited satellite visibility. To address these challenges, we propose a novel weighting approach, referred to as the HK model, that enhances real-time GPS positioning performance by leveraging the variability of the signal-to-noise ratio (SNR), without requiring auxiliary sensors. Analysis of 24 h observational datasets collected across diverse environments, including open-sky (OS), city streets (CS), and urban canyons (UC), demonstrates that multipath-affected non-line-of-sight (NLOS) signals exhibit significantly greater SNR variability than direct line-of-sight (LOS) signals. The HK model classifies received signals based on the standard deviation of their SNR and assigns corresponding weights during position estimation. Comparative performance evaluation indicates that relative to existing weighting models, the HK model improves 3D positioning accuracy by up to 22.4 m in urban canyon scenarios, reducing horizontal RMSE from 13.0 m to 4.7 m and vertical RMSE from 19.5 m to 6.9 m. In city street environments, horizontal RMSE is reduced from 11.6 m to 3.8 m. Furthermore, a time-sequential analysis at the TEHE site confirms consistent improvements in vertical positioning accuracy across all 24-hourly datasets, and in terms of horizontal accuracy, in 22 out of 24 cases. These results demonstrate that the HK model substantially surpasses conventional SNR- or elevation-based weighting techniques, particularly under severe multipath conditions frequently encountered in dense urban settings.

## 1. Introduction

Over the past few decades, the position coordinates obtained from global positioning system (GPS) devices have found various applications in daily life. However, in hard-to-obtain GPS reception situations, such as urban canyons and narrow alleys, it is difficult to determine the user’s location because four satellites cannot be tracked or because of harsh multipath effects. As is well known from the principle of GPS positioning, at least four visible satellites are required to locate themselves. However, it is often impossible to acquire a sufficient number of satellites because of signal blockages caused by nearby buildings. Even when more than four satellites are visible in an urban canyon, there is a high possibility that some of the received signals are from satellites to which direct line-of-sight (LOS) visibility cannot be obtained. This phenomenon is called multipath, meaning that the GPS signals transmitted from the satellite arrive at the receiver antenna through multiple paths. These signals may be reflected by the ground surface or structures around the receiver [1]. According to [2], a multipath signal includes all signals affected by reflection, diffraction, and scattering.

In general, to obtain reliable positioning results, one should not use those measurements that were taken in low elevation angles or were bounced from nearby reflecting surfaces. These types of low-quality and corrupted signals deteriorate positioning accuracy. However, in a field with limited satellite visibility, low-quality observations are sometimes required to increase the number of visible satellites and improve satellite geometry. Another reason to include them is to reduce the time required to obtain position fixes, such as the time to first fix (TTFF). In urban canyons, where having the visibility of more than four satellites is not an easy task, one may not be able to determine his/her location if multipath-impacted signals are removed during positioning. Thus, reliable methods should be devised to handle multipath signals effectively.

The signal-to-noise ratio (SNR) and satellite elevation angle can be used as indices by which to evaluate the deterioration of the GPS signal quality due to the multipath effect. These indices work as criteria by which to decide which measurement should be selected for positioning and to allocate noise covariance or add weights to the observation. Several weight models have been devised and can be classified as follows: (1) elevation models based simply on the elevation angle of the observed satellite; (2) exponential and SIGMA models utilizing measured SNR values; and (3) modified or combined models of (1) and (2). Hartinger and Brunner [3] introduced the SIGMA-ε model, in which variances are determined by SNR measurements. Brunner et al. [4] developed the SIGMA-Δ model, which applies template functions of SNR predictions based on the satellite elevation angle. Wiser and Brunner [5] realized the limitations of the SIGMA model, modified it using the Danish method, and developed an extended SIGMA model. Li and Wu [6] optimized the parameters of an exponential model where weights were modeled as an exponential function of SNR and evaluated their performances with respect to the original SIGMA model. Tay and Marais [7] considered SNR and elevation angle together and validated the new scheme in an urban canyon environment.

Even though there are slight differences in terms of weighting formulas and the way weights are assigned, the previous studies listed in the previous paragraph adopted a common approach of assigning weights according to satellite elevation angles and SNR values. Thus, they work adequately in an open-sky environment where changes in SNR measurements are very smooth, and a higher elevation angle implies a lower multipath. In urban canyons, where the SNR values fluctuate irregularly owing to the multipath effect, these methods are not efficient. In addition, a higher elevation angle and SNR do not necessarily indicate fewer multipath effects in urban canyons. Thus, one needs to distinguish direct signals from multipath-corrupted signals and allocate different weights depending on the severity of the multipath effect.

Xi et al. [8] enhanced the ambiguity fixing success rate and reduced noise in position estimates by collecting SNR values over extended observation intervals, normalizing these values, and incorporating the results into stochastic modeling. However, their approach focuses primarily on the correlation between average SNR values and satellite elevation angles, without considering epoch-to-epoch fluctuations in SNR. Wen et al. [9] addressed multipath effects in positioning by detecting multipath-impacted satellites in real time. Their method normalizes the C/N0 values based on elevation angle and processes each navigation satellite constellation individually. Although this normalization is applied on a per-satellite basis, the underlying statistical model is derived from datasets collected under open-sky conditions. Consequently, its effectiveness on mobile platforms in urban areas, where buildings of varying heights often obstruct satellite visibility, remains limited.

Fang et al. [10] improved positioning accuracy by incorporating the standard deviation of the SNR, measured over an arbitrary period, alongside the variances obtained during the estimation process. Specifically, if the standard deviation falls below a predefined threshold, the corresponding satellite is excluded from the positioning solution. However, in urban canyon environments, where the number of visible satellites is often significantly reduced, discarding additional satellites might make continuous positioning infeasible. Kubo et al. [11] reviewed various satellite exclusion methods, such as applying SNR thresholds based on elevation angle [12], using the fluctuation range of C/N0 over a specified duration [13], and employing residuals generated during the positioning process [14], comparing their respective advantages and disadvantages. In [11], the threshold is determined by analyzing a continuous time series of C/N0 values. If the C/N0 value drops below this threshold, the corresponding satellite signal is excluded from the positioning solution for a certain period, even if subsequent C/N0 values exceed the threshold. Nevertheless, this approach is inherently limited in that it may further reduce the number of available satellites, which is particularly problematic in urban canyon environments [10].

In this study, we developed a novel weight model to enhance GPS positioning integrity in urban canyons, where the multipath signal excessively interferes with the direct satellite signal. First, the SNR variability and its repeatability according to the receiver type and three distinctive signal reception environments were analyzed. Based on these results, a new weight model was devised based on an exponential function of SNR variability. The signals were separated into direct and non-direct based on their fluctuation patterns and given different weights. Finally, the developed model was validated in various environments, and time-sequential positioning accuracies were evaluated to demonstrate its integrity and continuity.

## 2. Characteristics of SNR

The SNR is an index indicating signal strength, and it is high for a direct signal received at the receiver antenna without any interference and low for a multipath signal bounced from nearby obstructions. If the measured SNR is high, it can generally be considered a direct and multipath-free signal, whereas low SNR signals can be regarded as multipath-impacted signals [15,16,17]. In addition, as the elevation angle decreases, the signal path length increases, and its SNR value decreases. This is because the signal must pass through a longer path through the ionosphere and, in particular, the troposphere before it reaches the receiver antenna, and the signal strength diminishes during the process. These two characteristics hold regardless of the receiver type and user environment. Nevertheless, the specific patterns and magnitudes of the SNR values depend on the receiver type and environment.

**Figure 1:**
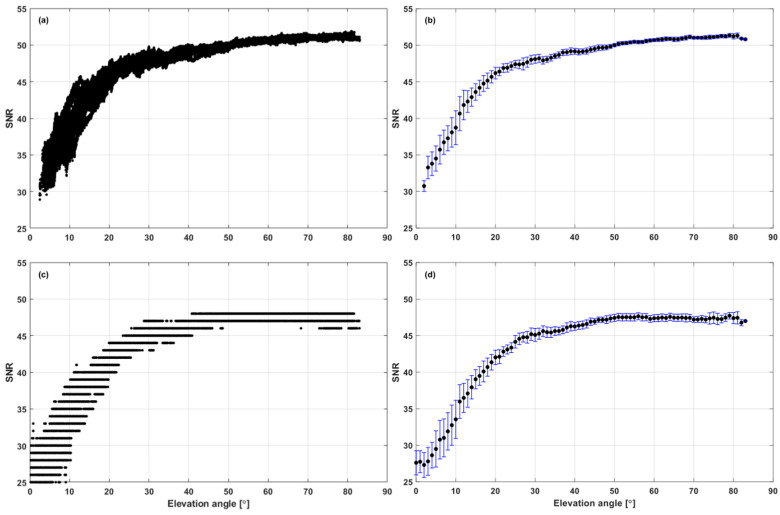
SNR values in dB-Hz, averages, and standard deviations at 1-degree intervals according to the receiver type: (**a**) NovAtel—SNR values against the elevation angle; (**b**) NovAtel—SNR averages and standard deviations against the elevation angle; (**c**) u-blox—SNR values against the elevation angle; (**d**) u-blox—SNR averages and standard deviations against the elevation angle.

To characterize the SNR according to the receiver type, we used temporary GPS equipment at Inha University, located in the city of Incheon, South Korea. One set of equipment consisted of a NovAtel OEMStar receiver and a NovAtel GPS-701GG antenna (NovAtel Inc., Calgary, AB, Canada), while the other set consisted of a u-blox EVK-5T receiver and a u-blox ANN-MS-0-005 patch antenna (u-blox AG, Zürcherstrasse, Switzerland). The data collection was conducted for four hours on DOY 296 (23 October 2013), and, during this period, the total number of satellites observed down to the horizon was 19. As we aimed to use the developed algorithm on mobile platforms, expensive geodetic-quality receivers and antennas were avoided. Figure 1a,c show the GPS L1 SNR values against the elevation angle, and Figure 1b,d show the averages and standard deviations at 1-degree intervals. It should be noted that the u-blox receivers output SNR values only in integers; thus, their plot contains vertical gaps, as shown in Figure 1c. As shown in Figure 1, the magnitudes and changing patterns of the SNR were slightly different between the two receiver antenna types. However, the general pattern of “high SNR for high elevation” holds in both cases. It is also notable that the variance was low at high elevation angles, and most of the signals observed below an elevation angle of 10° had SNR values lower than 40.

To investigate how the characteristics of the SNR values recorded at a GPS receiver change according to the signal reception environment, we collected data on a building roof, typical city streets, and urban canyons. Hereafter, we will denote a location without any signal obstruction above the elevation angle of 15° as OS, short for open sky. The worst hard-to-obtain GNSS reception location, such as metropolitan city streets with skyscrapers, is referred to as urban canyons (UC). Receivers located on typical city streets will be called CS, short for city streets. In our analysis, the OS case was chosen for the permanent IHU4 site located on the roof of a five-story building with clear satellite visibility in all directions. For the CS, a campaign-type station, SOND, was installed on the sidewalk of a four-lane street with five-to seven-story commercial buildings on both sides of the street. As a UC case, a campaign-type site named TEHE was placed in a parking lot on the southern side of Teheran-ro in Seoul. Teheran-ro is the street with the highest density of skyscrapers in South Korea, where one can obtain visibility from more than four GPS satellites for only 2–3 h during a day. The data was collected over 24 h at IHU4 and TEHE, and over three hours at SOND. The GPS equipment at IHU4 consisted of a Trimble NetRS receiver and Trimble Zephyr Geodetic II antenna, whereas a NovAtel OEMStar receiver and NovAtel GPS-701GG antenna were used at SOND and TEHE. The data span, including dates, in addition to the elevation cutoff angle, is as follows: (a) IHU4: 24 h on DOY 111 (21 April 2014), and the elevation cutoff angle is 10 degrees; (b) SOND: about three hours on DOY 245 (2 September 2014), and the elevation cutoff angle is five degrees; and (c) TEHE: 24 h on DOY 035 (4 February 2014), and the elevation cutoff angle is five degrees.

**Figure 2:**
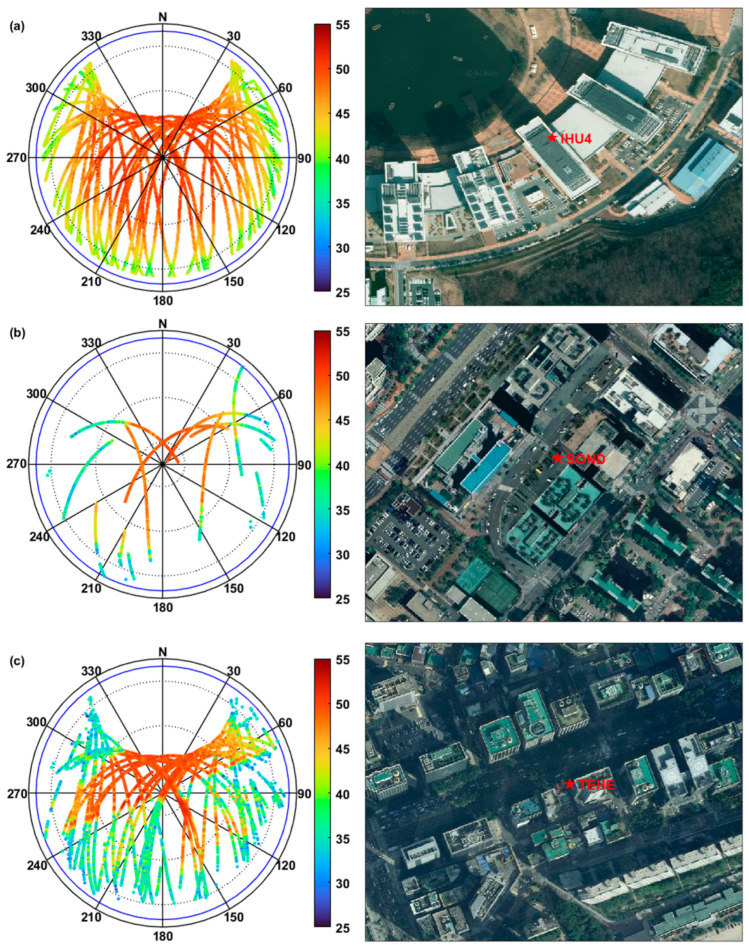
SNR values on a skyplot and aerial photographs by each observation environment: (**a**) open sky (IHU4); (**b**) typical city streets (SOND); (**c**) urban canyons (TEHE).

Figure 2 shows the SNR measurements at the three sites in a sky plot. To help determine the direction of the signal-blocking structures and understand their relation to the SNR observations, aerial photographs are also included in the figure. As shown in Figure 2a, the SNR is high at higher elevation angles, regardless of the azimuthal direction, and decreases as the elevation angle decreases. From Figure 2b,c on the contrary, one can see that SNR is high only along the street directions because the satellite visibility is maintained through the limited range of open sky along the street. In the cases of CS and UC, even those satellites whose visibility should be blocked by nearby buildings are observed, possibly through multiple paths, but show a very low SNR.

**Figure 3:**
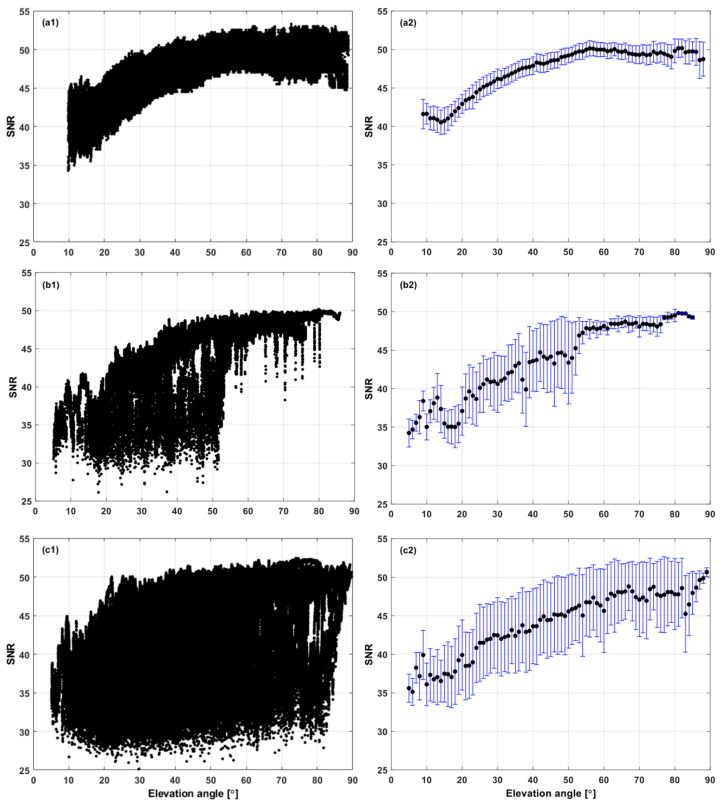
SNR values, averages, and standard deviations at 1-degree intervals according to the observation environment: (**a1**) IHU4—SNR values against the elevation angle; (**a2**) IHU4—SNR averages and standard deviations against the elevation angle; (**b1**) SOND—SNR values against the elevation angle; (**b2**) SOND—SNR averages and standard deviations against the elevation angle; (**c1**) TEHE—SNR values against the elevation angle; (**c2**) TEHE—SNR averages and standard deviations against the elevation angle.

The SNR variability was statistically evaluated in terms of the average and standard deviation according to the elevation angle. In Figure 3, the scatter plot of the SNR with respect to the elevation angle shows distinctive features. As the satellite visibility worsens, the standard deviation increases. Under the OS environment at IHU4, $\sigma_{SNR}$ is not dependent on the elevation angle at all and varies in the range of 0.8~2.4 dB-Hz. Meanwhile, at SOND, $\sigma_{SNR}$ remains nearly constant at 0.7 when the elevation angle is higher than 55° but becomes quite variable below it. This characteristic matches the height and density of the surrounding buildings at the site. Lastly, at TEHE, $\sigma_{SNR}$ fluctuates tremendously in all elevation angles except for the zenith direction of 80–90°. As mentioned before, a larger variability of $\sigma_{SNR}$ means the site is more affected by the multipath effect.

From the analysis of the behavior of the SNR variability patterns according to the signal-reception environment classified as OS, CS, or UC, we found that the magnitude and standard deviation of the SNR were highly dependent on the environment. Repeating patterns on two consecutive days can be used to prove that SNR variations are not determined by chance and are closely related to the environment. According to [18,19], multipath and phase center variations are the two most well-known error factors repeating on a sidereal day basis. Kim et al. [20] used satellite repeat times to show that the SNR patterns repeat exactly when the satellite returns to the same location in the sky after a sidereal day. Thus, it was concluded that the SNR is a function of the satellite geometry and the signal-reception environment and can thus be utilized as a criterion by which to determine whether the signal is affected by the multipath.

## 3. Typical Weight Models

As noted earlier, the SNR can be an effective tool for evaluating signal quality. In addition to the SNR, the satellite elevation angle was considered an index by which to weight GPS measurements. In this section, we review typical weight models based on the SNR and elevation angles and show how the new weight model was devised.

### 3.1. Elevation Model

As with a typical elevation model, one simply assigns a weight according to the satellite elevation angle ε by taking a certain sinusoidal function as in Equation (1). In this equation, the variance decreases as the elevation angle increases. In an extended Kalman filter (EKF), a diagonal matrix composed of those $\sigma_{i}^{2}$ is used as a measurement noise matrix [7].

(1) $$\sigma_{i}^{2}=\frac{1}{{sin}^{2}\left(\varepsilon_{i}\right)}$$

An advantage of this model is that it is simple; however, it cannot consider irregular situations where SNR measurements are low, even for high-elevation satellites, owing to multipath interference.

### 3.2. Exponential Model

In the model suggested by [6], the weights are given as exponential functions of the SNR. Equation (2) shows the typical implementation of an exponential model.

(2) $$\sigma_{i}^{2}=a+b\cdot e^{k\left(SNR_{min}-SNR_{i}\right)}$$

In Equation (2), $SNR_{min}$ and $SNR_{i}$ represent the preset minimum possible SNR and SNR measurements at the *i*-th epoch, respectively. Parameters *a*, *b*, and *k* should be empirically determined according to the receiver hardware and signal-reception environments. The positioning sensitivities of these three parameters were examined, and it was found that changing them did not have a significant effect on the positioning accuracy. Thus, we used the same values as those suggested by [6], for example, *a* = 0, *b* = 1, *k* = 0.3.

### 3.3. SIGMA Model

The SIGMA models were suggested by [3,5,6,21]. A typical representation of the SIGMA model is given by Equation (3), in which parameters *a* and *b* are empirically determined. In this study, we selected the values of *a* = 0 and *b* = 1.

(3) $$\sigma_{i}^{2}=a+b\cdot 10^{-0.1\cdot SNR_{i}}$$

### 3.4. SNR-Elevation Model

The weight models described in Section 3.1, Section 3.2 and Section 3.3 are given as functions of only the SNR or elevation angle. However, in the SNR-elevation model, these two factors were considered simultaneously. This approach was first suggested by [7], and its typical formula is given by Equation (4). Tay and Marais [7] recommended choosing the value of parameter *k* differently depending on the signal LOS analysis results. They used *k* = 1 when the signal was received directly from the satellite and assigned values other than 1 to the multipath-impacted signal. Thus, a larger variance must be allocated to the multipath signals.

(4) $$\sigma_{i}^{2}=k\cdot \frac{10^{-0.1\;\cdot \;SNR_{i}}}{\sin^{2}\left(\varepsilon_{i}\right)}$$

## 4. A New Weight Model Based on SNR

In Section 2, we confirmed that the magnitude and variability of the SNR change significantly depending on the signal-reception environment, even when using the same type of receiver and antenna. Especially on city streets and inside urban canyons, where GPS signals are severely affected by multipath errors, the standard deviations of SNR observables show relatively high values. Thus, the traditional weight models introduced in Section 3 are not effective in situations with multiple signal paths. This is because multipath errors can corrupt high-elevation angle measurements, and large SNR values can be observed for multipath-contaminated signals.

It is necessary to separate the direct signals from the multipath signals and assign appropriate weights accordingly. We refer to the direct signal from a visible satellite as LOS, and the indirect or multipath signal as NLOS (Non-LOS). To enhance positioning capabilities in CS and UC environments, a new criterion should be established to distinguish between LOS and NLOS and then allocate optimal weights based on these decisions.

To analyze the SNR characteristics of LOS and NLOS signals, we first determined whether the received signal was LOS or NLOS. This can be accomplished by conducting a satellite visibility analysis with 3D building models. If an observable is recorded on a receiver, we can compute the satellite position at that moment and derive an LOS vector that connects the user’s location to the satellite. If 3D geospatial information is available for nearby buildings, the signal blockage can be determined based on a visibility collision analysis. The LOS collision analysis utilizes a straight-line-polygon collision detection algorithm, which is widely used in fields such as 3D physics engines and graphics programming. In this context, the straight line refers to the LOS vector connecting an observation point and a satellite, and the polygon represents a building surface situated between the user and the satellite. The straight-line-polygon collision detection process is performed in three steps: (1) checking whether the two points that define the straight line lie on opposite sides of the polygon; (2) calculating the intersection point of the straight line and the plane that defines the polygon; and (3) checking whether the intersection point of the line and the plane lies within the area of the polygon. A detailed mathematical explanation can be found in [22].

**Figure 4:**
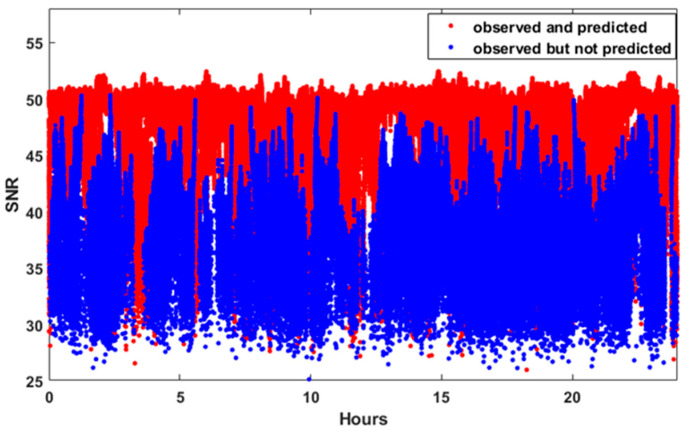
SNR measurements from all GPS satellites observed for 24 h at TEHE and comparison of LOS and NLOS signals.

Figure 4 shows the SNR measurements for all the GPS satellites observed for 24 h at TEHE, whose location corresponds to the UC case. In Figure 4, blue dots denote “predicted-and-observed” LOS signals, while red dots denote “not-predicted-but-observed” NLOS signals. The term “predicted” refers to the case in which GPS signals should be recorded because no signal blockage is anticipated. From the figure, it can be observed that the SNR values are significantly larger for the LOS signals.

**Figure 5:**
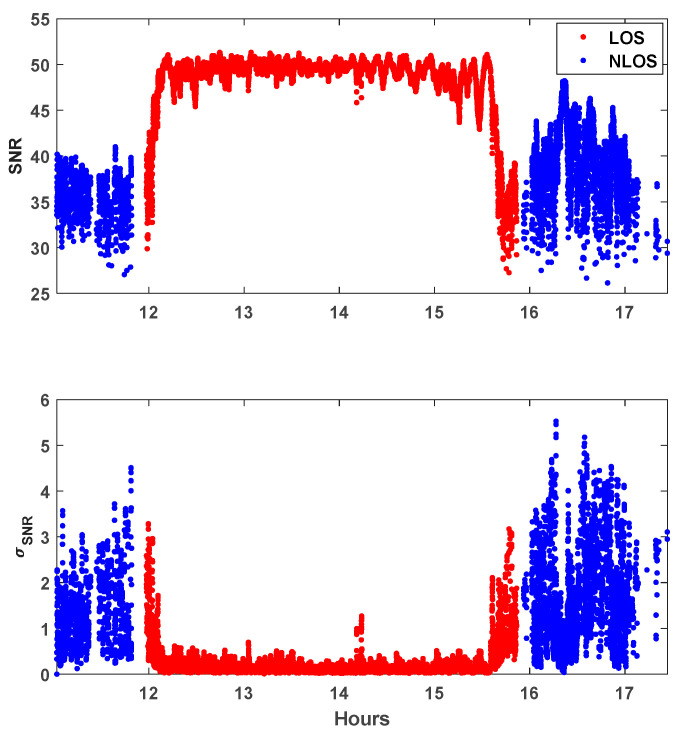
Time series of SNR and $\sigma_{SNR}$ of PRN 16 at TEHE and comparison of LOS and NLOS signals.

**Figure 6:**
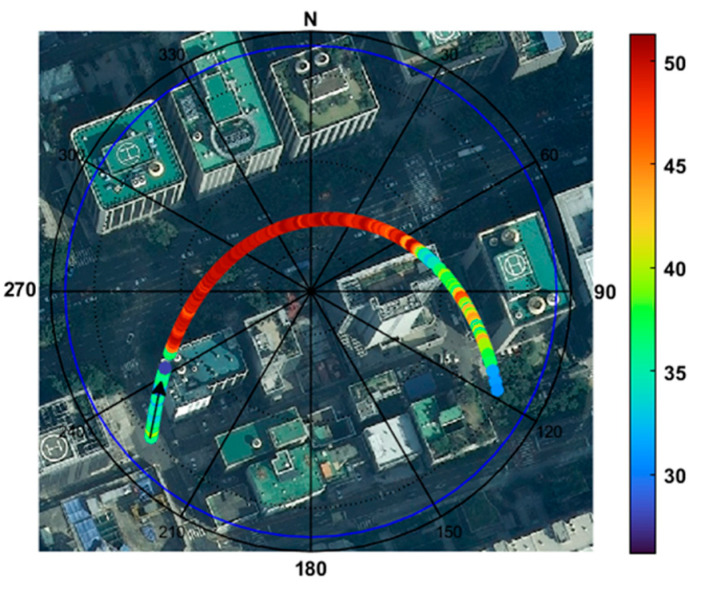
SNR observables of the PRN 16 depicted in a skyplot. The arrow indicates the direction of movement of PRN 16.

Figure 5 shows the time series of the SNR observables, with their standard deviations of PRN 16 at TEHE. Ten immediately preceding consecutive values have been used to compute the standard deviation of SNR at the final tenth epoch. PRN 16 was chosen because this satellite provides a representative mix of both LOS and NLOS signals. To compare the SNR values with their variability owing to surrounding buildings, Figure 6 shows the SNR observables of PRN 16 in a skyplot overlaid with an aerial photograph. In Figure 5, the blue and red dots depict the LOS and NLOS, respectively. While the SNR changes smoothly in an open-sky environment as the elevation angle increases or decreases, rapid fluctuations are observed at TEHE because of signal blockage by buildings existing in the line of sight of the satellite trajectory. It should be noted that NLOS measurements generally have a lower magnitude and higher variability than LOS signals. These unpredictable behaviors necessitate a new approach for separating LOS and NLOS signals and assigning appropriate weights accordingly.

The developed model is expressed by Equation (5) and is referred to as the HK model. In the equation, $\sigma_{SNR}$ is the standard deviation of ten consecutive SNR values and $SNR_{min}$ is the minimum SNR value. The parameter $SNR_{min}$ should be fixed in each data processing step but can be modified when the equipment or environment changes. Other parameters—*A*, *B*, α, and β—were empirically determined in this study. The model equation is a form of an exponential function of the standard deviation $\sigma_{SNR}$, and the weight will be chosen depending on the magnitude of $\sigma_{SNR}$ with the dividing criterion value of 1.0.

(5) $$\left\{\begin{array}{c} \sigma_{SNR}>1.0\;:\;\;\;\;\sigma_{i}^{2}=A\cdot e^{\alpha (SNR_{min}-{SNR}_{i})}\\ \sigma_{SNR}\leq 1.0\;:\;\;\;\sigma_{i}^{2}=B\cdot e^{\beta (SNR_{min}-{SNR}_{i})} \end{array}\right.$$

Here, NLOS and LOS signals were distinguished from the threshold value of 1.0, which was determined based on $\sigma_{SNR}$ analysis results for LOS/NLOS signals from all satellites observed at TEHE. Caution must be exercised because NLOS signals can be falsely identified as LOS signals if the threshold value is excessively high. By contrast, LOS signals can be misinterpreted as NLOS signals if the threshold value is too low. The mean $\sigma_{SNR}$ of NLOS signals from all satellites observed at TEHE was calculated as 0.8 ± 0.3. The threshold value was optimized by trying out values within the standard deviation. Specifically, seven threshold values were tested from 0.5 to 1.1, equally spaced at intervals of 0.1. A threshold value of 1.0 was the most effective choice for separating LOS from NLOS signals. Because this threshold is determined empirically, it can be changed. The same holds for the other model parameters, *A*, *B*, α, and β.

**Figure 7:**
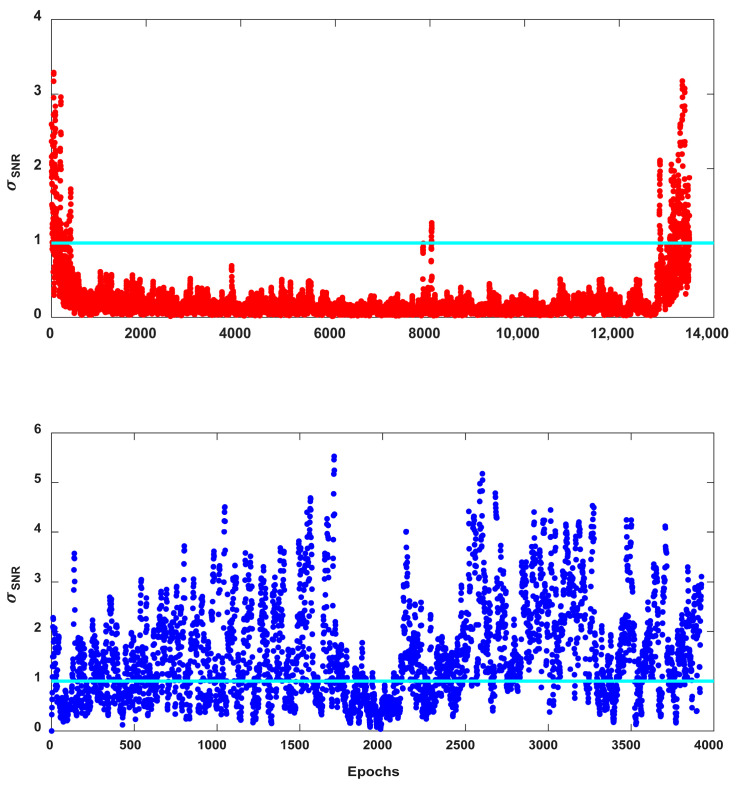
$\sigma_{SNR}$ values of the LOS and NLOS signals for PRN 16 at TEHE. The straight horizontal line in cyan denotes threshold value of 1.0. The red and blue dots depict the LOS and NLOS, respectively.

Figure 7 shows $\sigma_{SNR}$ computations of the LOS and NLOS signals for PRN 16, with the threshold value of 1.0 denoted as the straight horizontal line in cyan. The horizontal axis corresponds to the sequential numbering assigned separately to LOS and NLOS signals and does not reflect the time sequence of the entire measurement set. Values of $\sigma_{SNR}$ for LOS signals shown in the upper panel of Figure 7 were 0.5 or lower, except at the switching boundary between the LOS and NLOS signals. Most of the standard deviation values for the NLOS signals, depicted in the lower panel of Figure 7, remained above 0.5. However, LOS and NLOS signals cannot be clearly distinguished with one threshold for all satellites, and one should come up with different thresholds for each satellite. The difference in the threshold values may be due to errors in the building coordinates used for visibility analysis or unknown factors.

**Figure 8:**
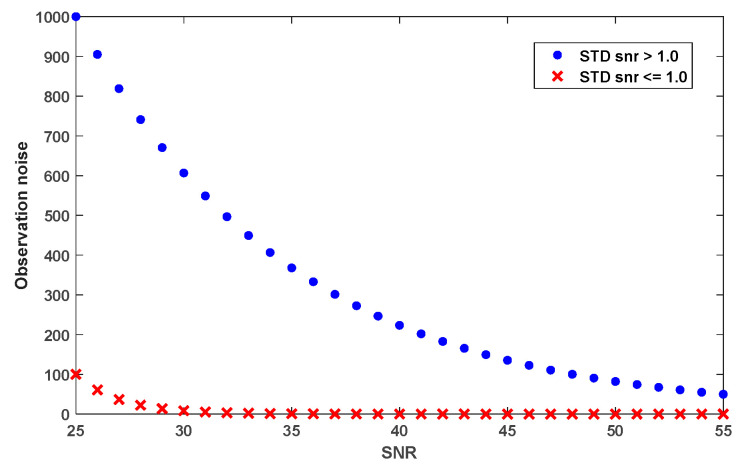
Measurement noise values from the HK model.

The simulated measurement noise of the HK model, as defined by Equation (5), is depicted in Figure 8 as a function of SNR values ranging from 25 to 55. The horizontal axis represents the SNR values, and the solid blue circles and red crosses correspond to NLOS and LOS signals, respectively. This clear differentiation between NLOS and LOS signals indicates that the HK model equation assigns much less weight or greater measurement noise values to NLOS signals than to LOS signals. By making this adjustment in the EKF, we can minimize the influence of NLOS signals.

**Figure 9:**
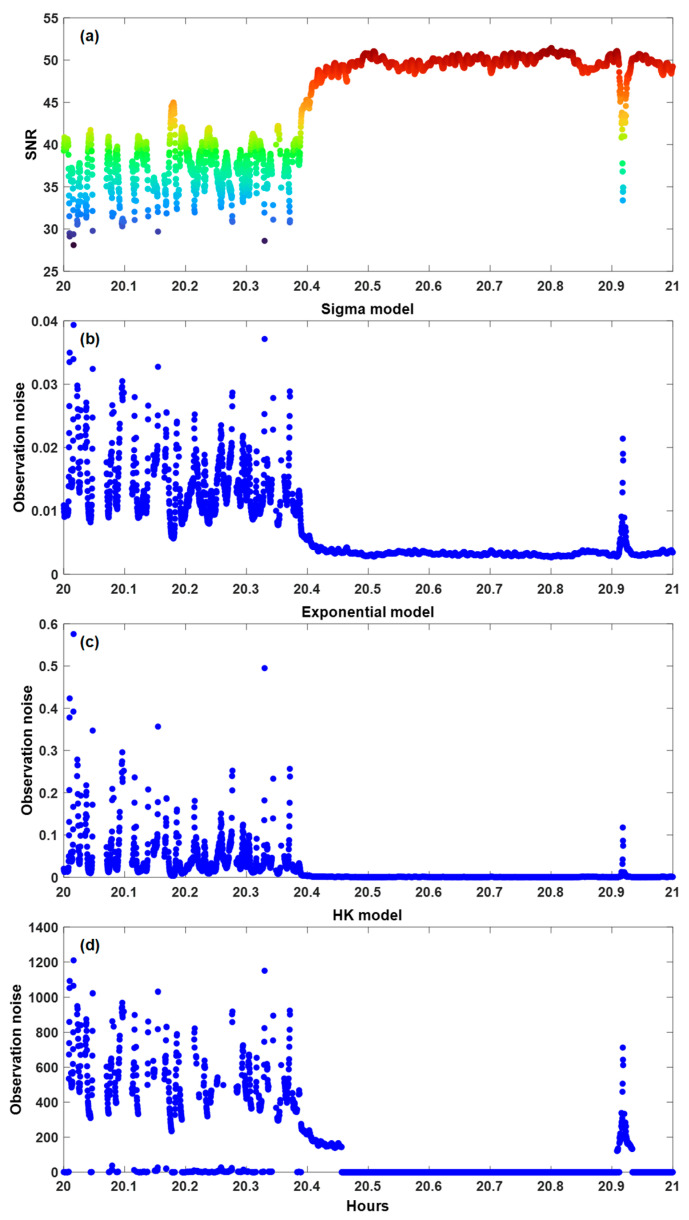
Time series of SNR values and measurement noises of PRN 17 for three different weight models: (**a**) SNR value of PRN 17; (**b**) the measurement noise using the sigma model; (**c**) the measurement noise using the exponential model; (**d**) the measurement noise using the HK model. The color of the dots refers to the color bar in Figure 6.

The variations in the measurement noise due to the three different SNR-based model equations are presented in Figure 9. For this analysis, PRN 17 is considered instead of PRN 16, which was examined in the preceding discussion. Furthermore, PRN 18 serves as an additional example that demonstrates a well-balanced integration of LOS and NLOS signal measurements. Figure 9a shows the SNR value of PRN 17, one of the satellites used for TEHE data processing, where the horizontal axis is the observation time, and the vertical axis denotes the SNR values. The subsidiary graphs in Figure 9b–d illustrate the measurement noise calculated from the SNR values in Figure 9a using the sigma, exponential, and HK models, respectively, with the vertical axis indicating the measured noise. Although the sigma model in Figure 9b determines the measurement noise by distinguishing between the LOS and NLOS signals, the values have a small range between zero and 0.04. Specifically, the differences in the measurement noise between the LOS and NLOS signals were not noticeable; hence, data processing was barely affected. The exponential model shown in Figure 9c has a range of measurement noise that is more than ten times higher than that produced by the sigma model. However, the exponential model cannot clearly distinguish between LOS and NLOS signals. The HK model can separate NLOS signals and produce noticeably high measurement noise values for the NLOS signals.

## 5. Validation Tests

The performance of the developed model was validated by comparing the positioning accuracies of the HK model with those of existing weight models. Accuracy evaluation was performed for the three sites. As noted in Section 2, 24-hourly datasets were collected for IHU4 and TEHE, while three hourly sets were collected for SOND. All datasets were sampled at 1 s intervals.

The positioning accuracy was compared by setting the true coordinates as those obtained from the online data processing service of the CSRS-PPP (Canadian Spatial Reference System-Precise Point Positioning). For more information on the CSRS-PPP, please see the Natural Resources Canada (NRCan) homepage [23]. According to [24], the horizontal accuracy of the CSRS-PPP service is ±(1–4) mm and the vertical accuracy is ±(2–7) mm. Gakstatter [25] indicated that the difference between the CSRS-PPP service and other online data processing services, such as the Australian Surveying and Land Information Group (AUSLIG) Online GPS Processing System (AUSPOS), GNSS Analysis and Positioning Software (GAPS), and the Online Positioning Users Service (OPUS), was insignificant on a millimeter scale.

### 5.1. Accuracy Analysis for Different Observation Environment

**Figure 10:**
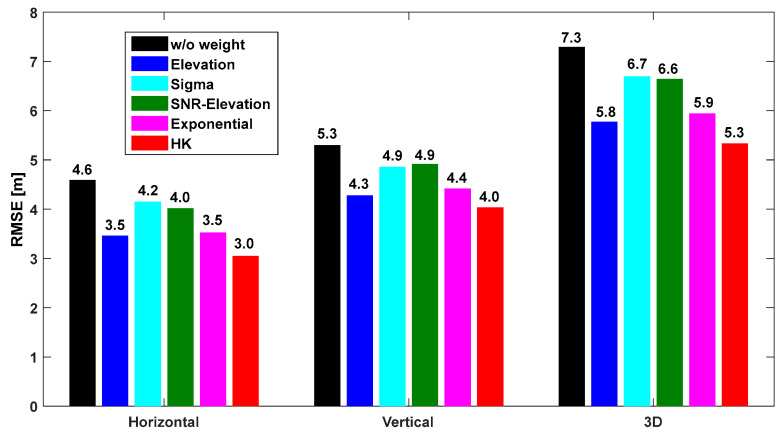
Horizontal, vertical, and 3D positioning RMSE according to the weight model at IHU4 shown in Figure 2a.

**Figure 11:**
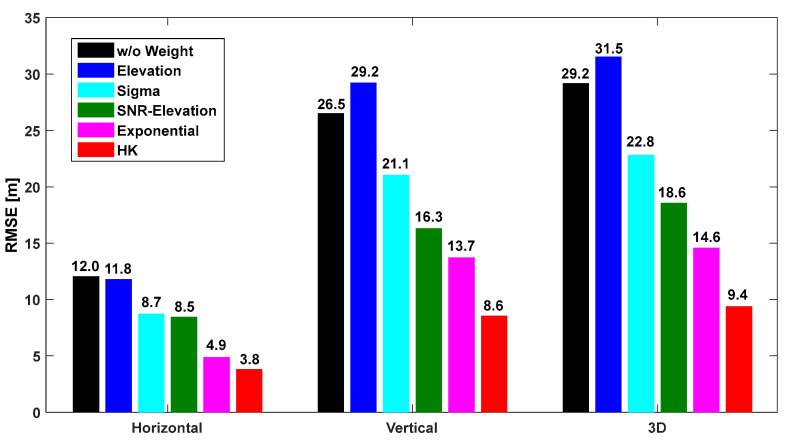
Horizontal, vertical, and 3D positioning RMSE according to the weight model at SOND shown in Figure 2b.

**Figure 12:**
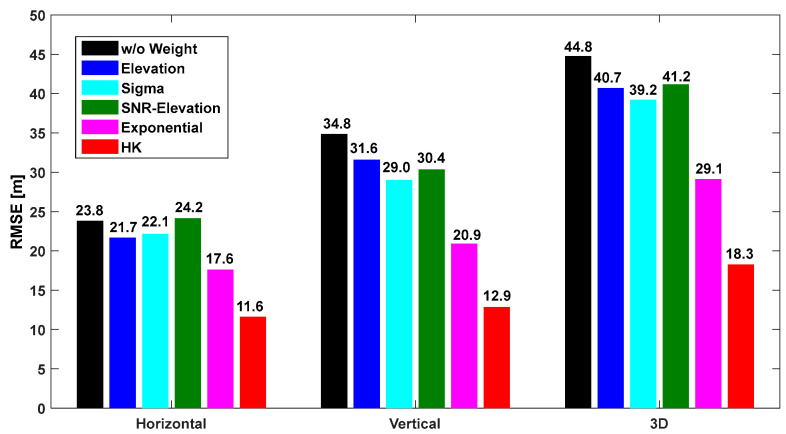
Horizontal, vertical, and 3D positioning RMSE according to the weight model at TEHE shown in Figure 2c.

The horizontal, vertical, and 3D root mean square error (RMSE) were calculated relative to the positions provided by CSRS-PPP. The mean RMSE values of the positioning errors were computed using the six weighted models. Figure 10, Figure 11 and Figure 12 show the results for the OS, CS, and UC cases, respectively. In the figures, “w/o weight” refers to the case where no weight was applied, and “HK model” refers to the model developed in this study. As shown in Figure 10, Figure 11 and Figure 12, the HK model significantly improved the accuracy compared with the other existing models. In the case of IHU4, as shown in Figure 10, the horizontal and vertical errors without a weight model were 4.6 m and 5.3 m, respectively. However, after applying the HK model, the horizontal and vertical errors were 3.0 m and 4.0 m, respectively, showing increased positioning accuracy. Moreover, the accuracy improved by at least 30 cm compared to the other models.

For the SOND site, as shown in Figure 11, the accuracy was three times better using the HK model compared to the “w/o weight” case; the horizontal RMSE was only 3.8 m, which was similar to the accuracy at IHU4. The HK model developed in this study outperformed other models. The results for the TEHE site are shown in Figure 12, indicating that the accuracy was two to three times better when the developed model was used compared to the “w/o weight” case. The accuracy improved by more than 10 m, indicating that the multipath effect could be significantly reduced. Because the weight was derived based on the SNR, which reflects the characteristics of multipath signals, the model is more effective when it is used for urban canyons rather than for the open sky. Among existing models, the exponential model exhibits the highest accuracy. Moreover, the SNR-based models were more effective than the elevation-angle-based models as the observation environment worsened.

### 5.2. Accuracy Analysis for Temporally Sequential Datasets

**Figure 13:**
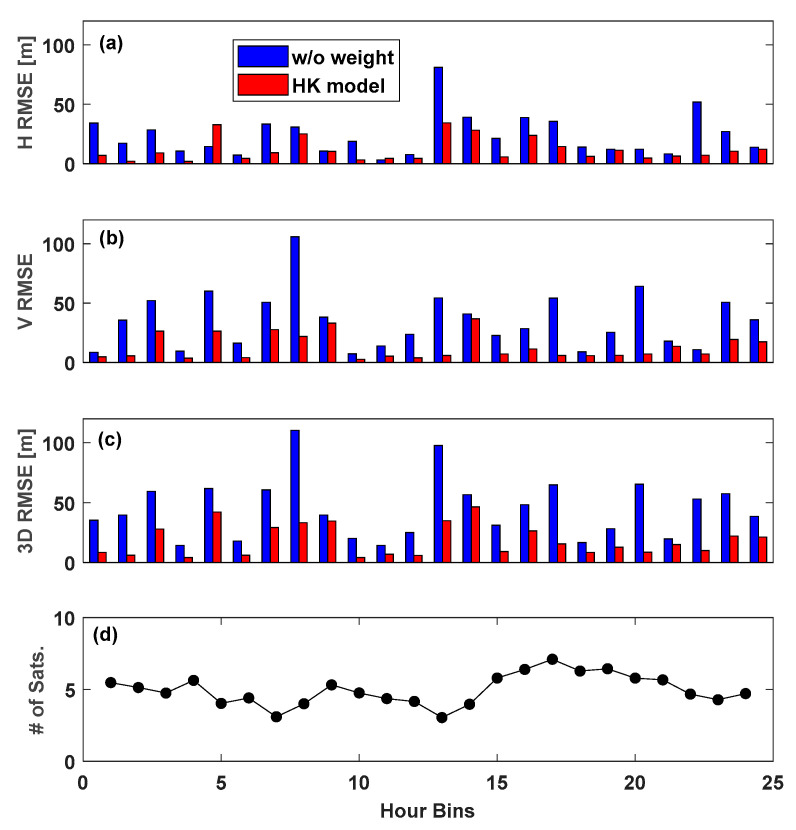
Improvement of positioning accuracies based on the HK weight model for 24-hourly datasets at TEHE in addition to the number of observed satellites: (**a**) hourly horizontal RMS error; (**b**) hourly vertical RMS error; (**c**) hourly 3D RMS error; (**d**) hourly number of visible satellites.

In this section, we present a detailed analysis of the TEHE site, which experienced the most severe multipath errors among the three cases described previously. Daily data received continuously for 24 h at TEHE were divided into 1 h sets, and hourly variations in positioning errors were examined. Figure 13a–c display the hourly horizontal, vertical, and 3D RMSEs resulting from applying the HK model to the TEHE site data. Figure 13 confirms that applying a weight using the HK model significantly improves all horizontal, vertical, and 3D accuracies. However, among the 24 datasets, the data for the fifth and eleventh hours showed unusual phenomena, with reduced accuracy in the horizontal direction. Nonetheless, 3D accuracy improved for all 24 datasets.

In the test results shown in Figure 13, the 3D positioning accuracies improved for every hourly dataset. However, the corresponding RMSE values varied significantly, ranging from the best case of 4.1 m to the worst error of 46.4 m. To relate this phenomenon with satellite availability, the average number of visible satellites each hour is shown in Figure 13d, from which one can see that a lower number of visible satellites resulted in decreased accuracy. The correlation coefficient between the number of visible satellites and the 3D RMSE was 0.44, indicating a positive correlation. In summary, the positioning accuracy was two to three times better than that of the HK model. However, model performance declined when fewer satellites were visible.

## 6. Conclusions

The HK model, a novel SNR-based weighting strategy proposed in this study, improves GPS positioning accuracy in multipath-prone environments such as city streets and urban canyons, outperforming conventional methods. By leveraging the standard deviation of SNR values, the model distinguishes between LOS and NLOS signals and assigns weights accordingly. Validation using observational datasets from three representative environments—open sky (OS), city street (CS), and urban canyon (UC)—demonstrated the superiority of the HK model over conventional elevation- or SNR-based weight models.

In the UC environment (TEHE site), where multipath interference was most severe, the HK model reduced the horizontal RMSE from 13.0 m to 4.7 m and the vertical RMSE from 19.5 m to 6.9 m, yielding a 3D positioning improvement of up to 22.4 m. On typical city streets (SOND site), horizontal RMSE improved from 11.6 m to 3.8 m, while at the OS site (IHU4), horizontal and vertical errors decreased from 4.6 m and 5.3 m to 3.0 m and 4.0 m, respectively. Temporal analysis at the TEHE site over 24-hourly intervals further confirmed the model’s reliability: 3D RMSE improved in all 24 cases, with horizontal improvements observed in 22 of 24 cases. The HK model consistently outperformed five other weight models, particularly under degraded satellite visibility conditions. These results demonstrate the model’s capability to enhance real-time GPS positioning accuracy by effectively mitigating multipath effects without the use of auxiliary sensors. The HK model includes several parameters that can be tailored empirically, allowing for optimization based on receiver manufacturer or hardware specifications. As a result, applying the model to different devices or environments may benefit from recalibrating these parameters to achieve optimal performance.

## Data Availability

The data should be available upon request to the corresponding author.

## Abbreviations

GPS	Global Positioning System
SNR	Signal to Noise Ratio
LOS	Line-of-Sight
NLOS	Non-Line-of-Sight
OS	Open Sky
CS	City street
UC	Urban Canyon
EKF	Extended Kalman Filter
PRN	Pseudo Random Number
HK	The new model, named after the first author’s initials
RMSE	Root Mean Square Error
